# Copper, cuproptosis, and cancer: biology concepts of a novel cell death

**DOI:** 10.1007/s10495-026-02432-w

**Published:** 2026-09-03

**Authors:** Reza Eshraghi, Nastaran Hosseini, Mohammad Sepehr Yazdani, Shirin Badihi, Pedram Pirmoradian, Ashkan Bahrami, Bahar Darouei, Shayan Ghanouni, Alfred K. Lam, Michael R. Hamblin

**Affiliations:** 1 Social Determinants of Health Research Center, Isfahan University of Medical Sciences, Daneshgah Bvld, Isfahan, 8174673441 Iran; 2 School of Medicine, Isfahan University of Medical Sciences, Isfahan, Iran; 3 School of Medicine, Kashan University of Medical Sciences, Isfahan, Iran; 4 School of Medicine and Dentistry, Griffith University, Gold Coast Campus, Southport, Gold Coast, QLD 4222 Australia; 5 Pathology Queensland, Gold Coast University Hospital, Southport, Gold Coast, QLD 4215 Australia; 6 Faculty of Health Science, Laser Research Centre, University of Johannesburg, Doornfontein, 2028 South Africa

**Keywords:** Cuproptosis, Copper homeostasis, Copper, Cancer treatment, Mitochondrial dysfunction

## Abstract

Copper is an essential trace element required for mitochondrial respiration, redox regulation, iron metabolism, and cellular signalling, but excessive or mislocalised copper can be cytotoxic. Cuproptosis is a recently identified form of regulated cell death in which copper binds to lipoylated mitochondrial proteins, promotes their aggregation, destabilises iron–sulfur cluster proteins, and induces mitochondrial proteotoxic stress. Copper therefore has context-dependent roles in cancer. Physiological copper supports tumour metabolism, angiogenesis, extracellular-matrix remodelling, and selected oncogenic signalling pathways, whereas therapeutic copper depletion can inhibit copper-dependent tumour processes. Conversely, copper ionophores and related approaches may increase intracellular copper sufficiently to induce cuproptosis in metabolically susceptible cancer cells. This review describes systemic and intracellular copper homeostasis, including intestinal absorption, intracellular trafficking, mitochondrial copper distribution, storage, and export. Particular attention is given to CTR1/SLC31A1, the functionally distinct copper-transporting ATPases ATP7A and ATP7B, metallothioneins, copper chaperones, and cytochrome c oxidase assembly factors. We also examine how cancer cells reprogramme copper handling to support proliferation, angiogenesis, metastasis, and immune evasion. Finally, we discuss the molecular basis of cuproptosis, including the roles of FDX1, FDXR, LIAS, DLAT, mitochondrial respiration, protein lipoylation, and iron–sulfur cluster destabilisation. Current evidence indicates that cuproptosis susceptibility varies among tumour types and depends on copper handling, mitochondrial metabolic state, and the integrity of the protein-lipoylation machinery. Defining these determinants will be necessary for the development of tumour-selective copper-targeted therapies.

**Graphical abstract:**

**Figure:**
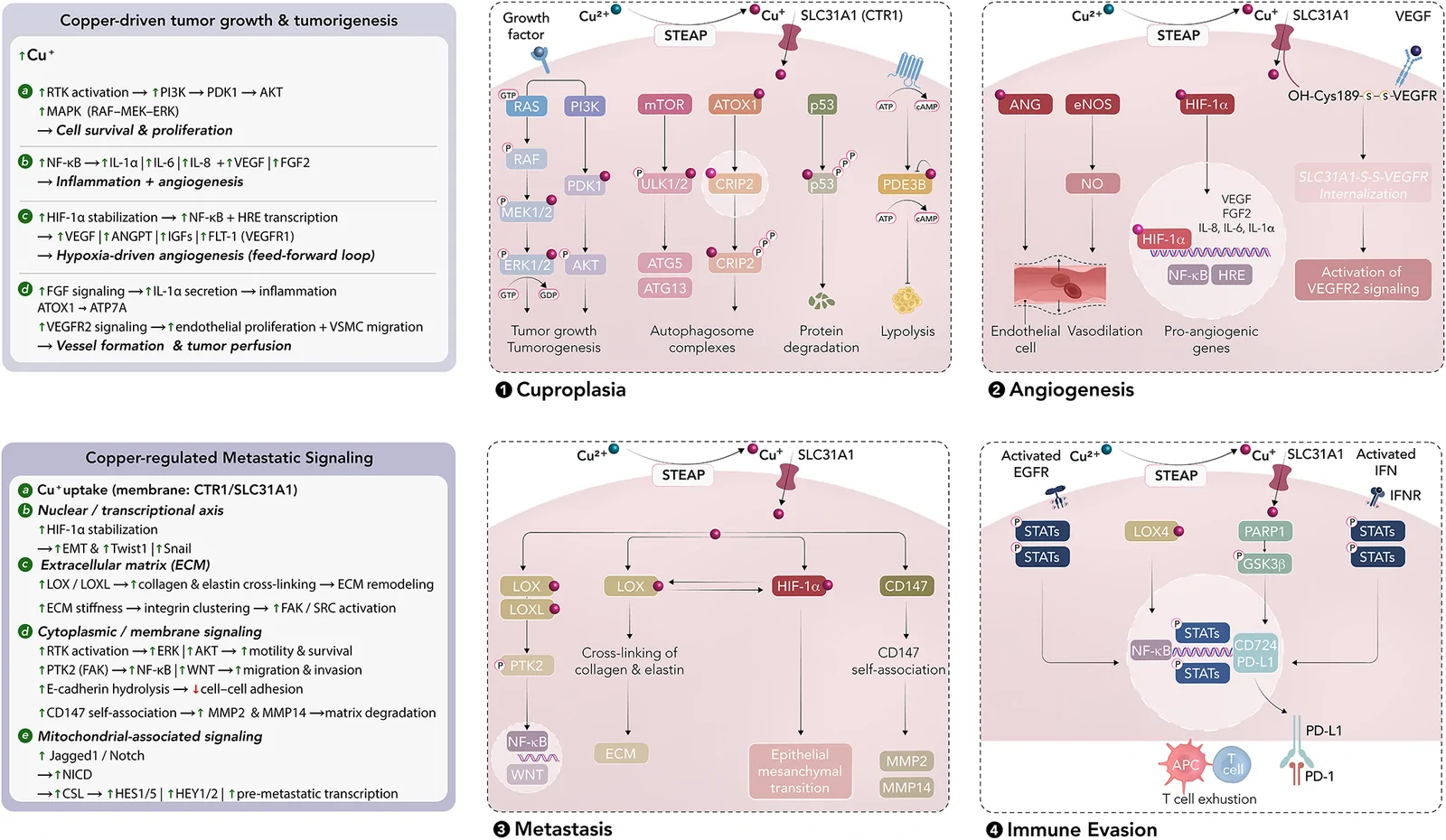

## Introduction

Copper is an essential trace element that supports key cellular processes, including mitochondrial respiration, redox balance, iron metabolism, angiogenesis, and signalling [1, 2]. Because excess copper can be toxic, cells tightly regulate its uptake, transport, storage, mitochondrial handling, and export. In cancer, copper metabolism is often disrupted in ways that promote malignant transformation and tumour progression [3]. Abnormal regulation of copper transporters and chaperones can cause copper to accumulate or redistribute within cancer cells, creating both a metabolic weakness and a potential therapeutic target. While copper has long been recognised as important in cancer biology, recent findings have shown that it can also directly induce a distinct form of regulated cell death [4].

Cuproptosis is a newly identified copper-dependent form of regulated cell death that is mechanistically distinct from apoptosis, ferroptosis, and other established death pathways [4]. Rather than being mainly driven by DNA fragmentation, membrane disruption, or lipid peroxidation, cuproptosis is strongly associated with mitochondrial metabolism. Cuproptosis is triggered when excessive intracellular copper binds to lipoylated proteins in the tricarboxylic acid (TCA) cycle, particularly a key mitochondrial enzyme, dihydrolipoamide S-acetyltransferase (DLAT), causing protein aggregation, depletion of iron–sulfur cluster proteins, mitochondrial proteotoxic stress, and ultimately cell death [4]. The FDX1–LIAS–DLAT axis is central to this process, linking copper redox activity, mitochondrial protein lipoylation, and metabolic susceptibility to cuproptosis [5].

The discovery of cuproptosis provides a new therapeutic opportunity in cancer, particularly for tumours with disrupted copper homeostasis or strong dependence on mitochondrial respiration. By intervening in copper transport, mitochondrial metabolism, or intracellular copper accumulation through ionophores [6–8], chelators [9, 10], transporter inhibitors [11], or nanomedicine platforms [12–14], cuproptosis-based strategies may selectively induce lethal mitochondrial proteotoxic stress in cancer cells. However, effective clinical translation requires a detailed understanding of tumour-specific copper metabolism, resistance mechanisms, and cuproptosis-regulatory pathways to maximise antitumour efficacy while minimising toxicity to normal tissues.

In this narrative review, we summarise the biological principles governing systemic and cellular copper homeostasis and examine their relevance to cancer. We describe the molecular mechanism of cuproptosis, with particular emphasis on mitochondrial respiration, FDX1 and FDXR, the lipoic-acid pathway, lipoylated TCA-cycle proteins, and iron–sulfur cluster destabilisation. We then evaluate how altered copper handling contributes to tumour growth and treatment response and assess the opportunities and limitations associated with copper ionophores, chelators, transporter modulation, and other copper-targeted strategies. This framework distinguishes established cuproptosis mechanisms from broader forms of copper toxicity and identifies the tumour-specific factors that may determine therapeutic susceptibility.

## Copper homeostasis

**Fig. 1:**
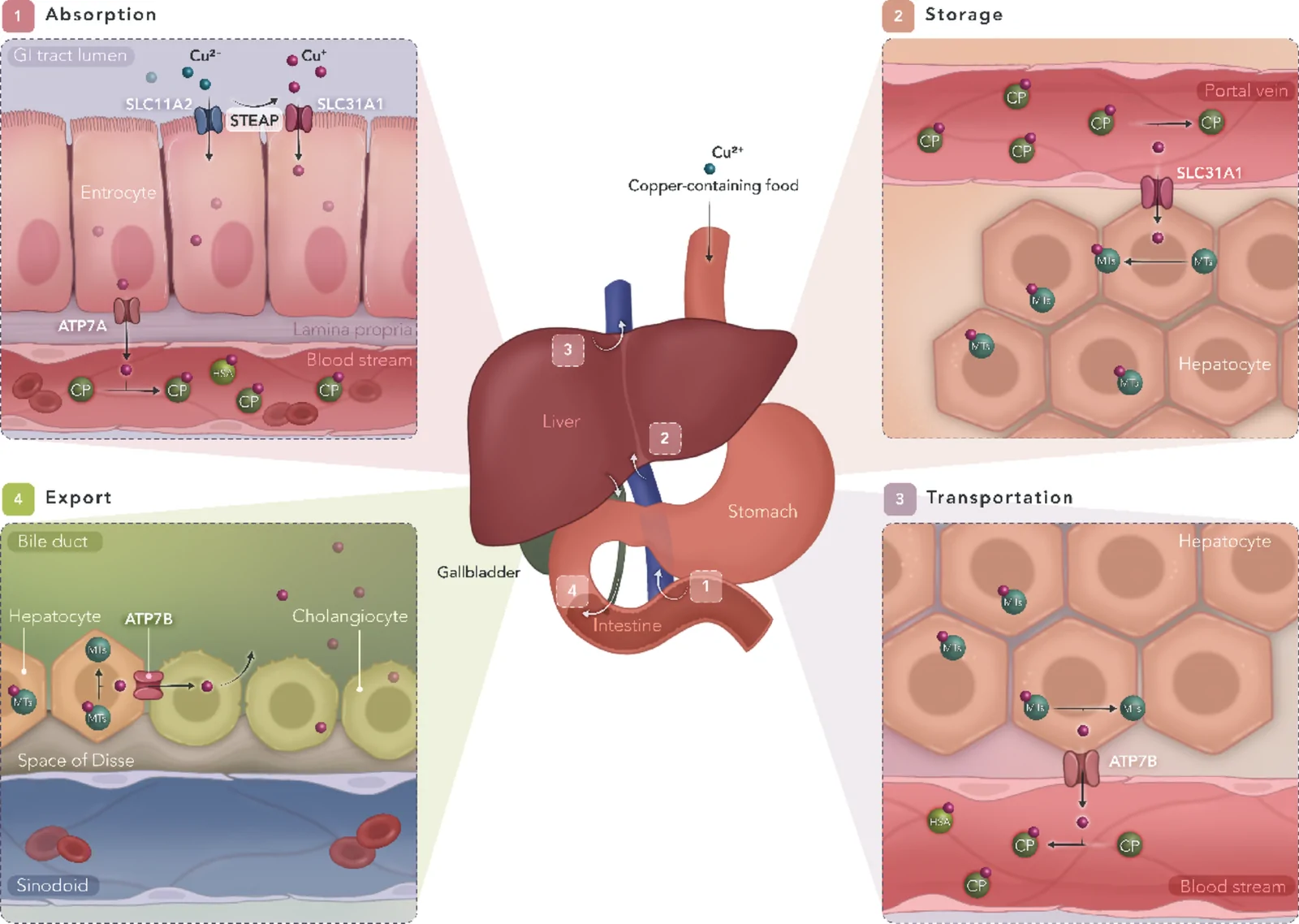
Systemic copper homeostasis in the GI tract: absorption, storage, transport, and export. The physiological regulation of copper (Cu) in four major compartments critical to copper metabolism. (1) Absorption. Dietary copper (primarily Cu^2^⁺) is absorbed in the small intestine. Cu^2^⁺ is reduced to Cu⁺ by STEAP proteins and transported into enterocytes via SLC31A1 (CTR1). ATP7A mediates basolateral Cu⁺ export from enterocytes into the portal circulation, where it is initially associated mainly with albumin, amino acids, and other low-molecular-weight ligands before hepatic uptake. (2) Storage. Hepatocytes take up circulating copper via SLC31A1 and store it bound to metallothioneins (MTs), serving as a buffer against both deficiency and overload. (3) Transportation. Within hepatocytes, copper is trafficked via ATP7B for incorporation into ceruloplasmin (CP), which is secreted into the bloodstream, distributing bioavailable copper to peripheral tissues. (4) Export. Excess copper is excreted into the bile through ATP7B-mediated transport in hepatocytes and cholangiocytes, thus preventing toxic accumulation. ATP7A: ATPase copper transporting alpha, ATP7B: ATPase copper transporting beta, SLC31A1 (CTR1): Solute Carrier Family 31 Member 1 (Copper Transporter 1), SLC11A2: Solute Carrier, Family 11 Member 2 (Divalent Metal Transporter 1), STEAP: Six Transmembrane Epithelial Antigen of the Prostate (copper reductase), CP: Ceruloplasmin, MT: Metallothionein, GI: Gastrointestinal

Copper is an essential micronutrient whose absorption, distribution, utilisation, storage, and excretion must be tightly regulated. Recommended dietary intake varies according to age, physiological state, and the guideline applied; therefore, any numerical intake range should be stated with reference to a specific nutritional authority. Excess copper can disrupt redox homeostasis and damage proteins, lipids, and nucleic acids, whereas copper deficiency impairs the activity of essential cuproenzymes [15]. Systemic copper balance is maintained primarily through intestinal absorption and hepatic storage and excretion. As illustrated in Fig. 1, dietary copper is absorbed by enterocytes, exported across the basolateral membrane mainly through ATP7A, transported to the liver, incorporated into ceruloplasmin through ATP7B-dependent secretory trafficking, or eliminated through ATP7B-mediated biliary excretion.

Copper also functions as a catalytic cofactor for enzymes involved in mitochondrial respiration, antioxidant defence, iron metabolism, extracellular-matrix maturation, neuroendocrine signalling, catecholamine synthesis, and pigmentation [16–18]. Representative copper-dependent enzymes include cytochrome c oxidase, Cu/Zn superoxide dismutase, ceruloplasmin, hephaestin, lysyl oxidase-family enzymes, and tyrosinase, as summarised in Table 1. Consequently, copper dysregulation can affect cancer biology through several distinct mechanisms, including altered cuproenzyme activity, redistribution of bioavailable copper, oxidative injury, and, under defined conditions, cuproptosis [19–22].

**Table 1:** Representative copper-dependent enzymes and their physiological roles

Copper-dependent enzyme	Main physiological role
COX/ complex IV	Catalyses terminal electron transfer from cytochrome c to molecular oxygen and contributes to the proton gradient that drives mitochondrial ATP synthesis [23, 24]
Superoxide dismutase (SOD)	Cytosolic Cu/Zn superoxide dismutase that converts superoxide into hydrogen peroxide and molecular oxygen, thereby limiting intracellular oxidative stress [25, 26, 27]
Ceruloplasmin	Plasma multicopper ferroxidase that carries most circulating copper and oxidises ferrous iron, supporting iron export and systemic iron trafficking [28]
Hephaestin	Intestinal multicopper ferroxidase involved in basolateral iron transfer from enterocytes into the circulation [29]
Lysyl oxidase family enzymes	Copper-dependent amine oxidases that cross-link collagen and elastin, thereby regulating extracellular matrix maturation, stiffness, and connective tissue integrity [30]
Tyrosinase	Copper-containing enzyme that catalyses tyrosine hydroxylation to L-DOPA and oxidation of L-DOPA to dopaquinone during melanin biosynthesis [31]

ATP: adenosine triphosphate, COX: cytochrome c oxidase, Cu: copper, Zn: zinc, SOD: superoxide dismutase, L-DOPA: L-3,4-dihydroxyphenylalanine

### Systemic copper metabolism: absorption, transport, and excretion

Systemic copper homeostasis requires coordinated intestinal absorption, protein-bound transport, hepatic uptake, storage, and biliary excretion [32]. Dietary copper is absorbed predominantly in the proximal small intestine. Its bioavailability can be modified by interactions with other dietary components, including iron and zinc [33]. Copper-rich foods include shellfish, organ meats, nuts, legumes, whole grains, and selected vegetables [34]. At the enterocyte surface, metalloreductases such as STEAP proteins and duodenal cytochrome b can reduce Cu^2^⁺ to Cu⁺, the preferred substrate for high-affinity copper uptake [35, 36].

Cu⁺ enters enterocytes primarily through copper transporter 1, also known as CTR1 or SLC31A1. ATP7A subsequently mediates basolateral copper export into the portal circulation [37]. Before hepatic uptake, circulating copper is associated with albumin, amino acids, and other low-molecular-weight ligands; most copper in mature plasma is carried by ceruloplasmin [22, 38]. Hepatocytes take up portal copper and buffer part of the intracellular pool through binding to metallothioneins and other ligands [39–41]. ATP7B transports copper into the secretory pathway for incorporation into ceruloplasmin and, when hepatic copper increases, traffics toward apical or canalicular compartments to facilitate biliary excretion [42]. Copper is subsequently distributed to peripheral tissues, including skeletal muscle, bone, and the nervous system [43]. Reported estimates of tissue copper distribution vary according to the population and measurement method; therefore, percentage values for individual compartments should be retained only if they are directly supported by an appropriate quantitative source [44].

### Intracellular copper transport and regulation

Intracellular copper homeostasis determines whether copper is safely incorporated into cuproenzymes, sequestered by buffering systems, exported from the cell, or accumulated in a form that can become toxic. Because cuproptosis depends on mitochondrial metabolism and the interaction of copper with lipoylated mitochondrial proteins, pathways controlling copper uptake, buffering, subcellular distribution, and efflux can influence cellular susceptibility [4, 37, 45–47].

#### Copper uptake and balance

CTR1/SLC31A1 is the principal high-affinity Cu⁺ importer at the plasma membrane and therefore has a central role in cellular copper acquisition [46, 48, 49]. After entry, copper is transferred among cytosolic ligands, metallochaperones, and intracellular compartments rather than existing as a substantial pool of freely solvated ions [50]. Most intracellular copper is tightly protein-bound, whereas a much smaller, kinetically accessible pool participates in copper trafficking and signalling [51]. Metallothioneins, glutathione, and dedicated copper chaperones restrict inappropriate copper reactions and help maintain the labile copper pool within a narrow physiological range [47, 52].

#### CCS, SOD1, and redox regulation

**Fig. 2:**
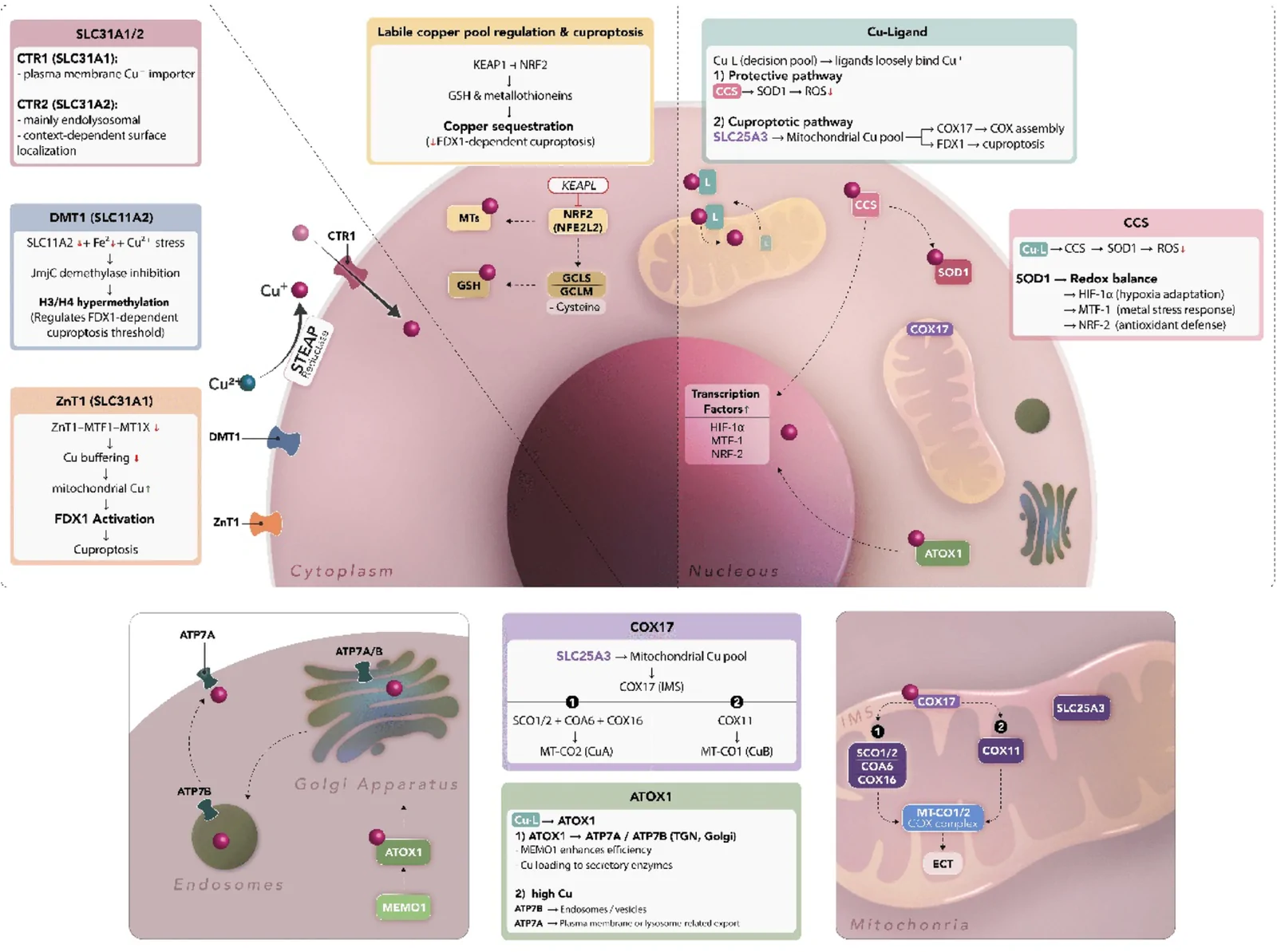
Regulation of the labile copper pool and its bifurcated roles in protective or cuproptotic mechanisms. Plasma membrane import of Cu⁺ is mediated by CTR1 (SLC31A1), while endosomal uptake may involve CTR2 (SLC31A2) and DMT1 (SLC11A2). Once inside the cytoplasm, Cu⁺ is either buffered by metallothioneins and glutathione (GSH) or transferred to Cu-ligands (Cu-L). In the protective pathway, Cu⁺ is delivered to SOD1 via CCS, maintaining redox balance through NRF2, MTF-1, and HIF-1α transcriptional activity. Alternatively, Cu⁺ can enter the mitochondria via the COX17–SLC25A3 axis, where it is directed toward cytochrome c oxidase (COX) assembly or, if dysregulated, promotes cuproptosis via FDX1 activation. ZnT1 (SLC30A1) and DMT1 modulate this bifurcation by controlling mitochondrial Cu⁺ levels and the chromatin state (via H3/H4 methylation). ATOX1 transfers cytosolic Cu⁺ to ATP7A or ATP7B at the TGN. ATP7A functions mainly in extrahepatic cells, where it supplies secretory-pathway cuproenzymes and can traffic toward peripheral vesicles or the plasma membrane to promote copper efflux. ATP7B functions predominantly in hepatocytes, where it supplies copper for ceruloplasmin metallation and traffics toward apical/canalicular compartments to support biliary copper excretion. The fate of copper, whether antioxidant protection or cell death, is finely tuned by the labile copper pool, redox ligands, and mitochondrial Cu⁺ handling machinery. CTR1 (SLC31A1): Copper Transporter 1, CTR2 (SLC31A2): Copper Transporter 2, DMT1 (SLC11A2): Divalent Metal Transporter 1, ZnT1 (SLC30A1): Zinc Transporter 1, FDX1: Ferredoxin 1, GSH: Glutathione, MTs: Metallothioneins, KEAP1: Kelch-like ECH-associated protein 1, NRF2 (NFE2L2): Nuclear Factor Erythroid 2–Related Factor 2, CCS: Copper Chaperone for SOD1, SOD1: Superoxide Dismutase 1, MTF-1: Metal Response Element–Binding Transcription Factor 1, HIF-1α: Hypoxia-Inducible Factor 1-alpha, ATOX1: Antioxidant 1 Copper Chaperone, ATP7A/B: ATPase copper transporters A and B, COX: Cytochrome c Oxidase, COX17: Cytochrome c Oxidase Copper Chaperone, SLC25A3: Mitochondrial Phosphate Carrier, SCO1/2, COA6, COX16: COX Assembly Factors, MEMO1: Mediator of Cell Motility 1, MS: Mitochondrial Space, CuA/CuB: Copper Centers A and B in COX

Copper chaperone for superoxide dismutase (CCS) delivers copper to superoxide dismutase 1 (SOD1) and facilitates the oxidative maturation required for its enzymatic activity [53–57]. SOD1 is located predominantly in the cytosol, with a smaller pool in the mitochondrial intermembrane space, where it catalyses the dismutation of superoxide into hydrogen peroxide and molecular oxygen [58, 59]. This activity limits superoxide accumulation but also generates hydrogen peroxide, which must subsequently be removed by catalase, peroxiredoxins, or glutathione-dependent antioxidant systems. As illustrated in Fig. 2, CCS-dependent SOD1 maturation therefore contributes to cellular redox homeostasis. Copper that is not immediately transferred to cuproproteins is buffered by metallothioneins, glutathione, and other intracellular ligands, thereby limiting inappropriate copper-mediated reactions (47).

#### Mitochondrial copper transport and cytochrome c oxidase assembly

Mitochondria require copper for the assembly and activity of cytochrome c oxidase, or respiratory-chain complex IV [60]. A soluble, low-molecular-weight copper–ligand pool has been identified in the mitochondrial matrix, although its chemical identity and physiological trafficking route remain incompletely defined [61, 62]. SLC25A3 participates in mitochondrial copper handling and cytochrome c oxidase biogenesis [24, 63, 64]. Earlier studies supported copper transport into the mitochondrial matrix, whereas more recent evidence also implicates SLC25A3 in the export of matrix copper for metallation of cytochrome c oxidase. Its direction of transport should therefore be described according to the experimental model rather than as exclusively inward or outward.

Cytochrome c oxidase contains two copper centres: the CuA centre in subunit COX2 and the CuB centre in subunit COX1 [65, 66]. Copper insertion into these sites requires a coordinated network of assembly factors in the mitochondrial intermembrane space. COX17 transfers Cu⁺ to SCO1/SCO2 and COX11-dependent pathways. SCO proteins participate in the maturation of the CuA centre in COX2, whereas COX11 contributes to CuB-centre formation in COX1 [67–72]. Disruption of this copper-delivery network impairs cytochrome c oxidase assembly and activity, leading to defective mitochondrial respiration and altered redox homeostasis [73].

#### Roles in the Golgi apparatus and nucleus

The trans-Golgi network (TGN) is a major intracellular site of copper distribution to secretory-pathway cuproenzymes [74]. ATP7A and ATP7B are homologous P-type copper-transporting ATPases that receive Cu⁺ from the cytosolic chaperone ATOX1 and transport it across trans-Golgi network or post-Golgi membranes [75]. Despite this shared biochemical function, ATP7A and ATP7B have distinct physiological roles and should not be considered interchangeable. ATP7A functions mainly in extrahepatic tissues. In enterocytes, it mediates basolateral copper export into the portal circulation; in other peripheral cells, it supplies copper to secretory-pathway cuproenzymes and supports cellular copper efflux. Under basal or copper-limited conditions, ATP7A is predominantly localised to the TGN. When intracellular copper rises, ATP7A redistributes toward peripheral vesicles and the plasma membrane, thereby facilitating copper export; it returns toward the TGN after copper concentrations decline [37, 76, 77]. ATP7B functions predominantly in hepatocytes, where it supplies copper for incorporation into ceruloplasmin and mediates the principal route of copper elimination through bile. Under basal conditions, ATP7B is mainly localised to the TGN. Increased intracellular copper promotes its redistribution toward apical/canalicular endolysosomal compartments that support biliary copper excretion, whereas copper lowering promotes retrieval of ATP7B toward the TGN [78–81].

Pathogenic variants in ATP7A cause Menkes disease, an X-linked disorder in which impaired basolateral copper export from enterocytes reduces copper delivery to peripheral tissues and produces systemic copper deficiency [77, 82]. The resulting impairment of copper-dependent enzymes contributes to progressive neurodevelopmental deterioration, seizures, connective-tissue abnormalities, growth failure, and characteristic sparse or kinky hair [83]. By contrast, biallelic pathogenic variants in ATP7B cause Wilson disease, an autosomal-recessive disorder characterised by defective biliary copper excretion and impaired copper incorporation into ceruloplasmin [78, 79]. Copper initially accumulates predominantly in the liver and may subsequently affect the brain and other organs, resulting in hepatic, neurological, and psychiatric manifestations [80, 81, 84].

ATP7A and ATP7B metallate different sets of secretory-pathway cuproproteins in a tissue-dependent manner. ATP7A supplies copper to several extrahepatic cuproenzymes, including lysyl oxidase-family enzymes and, in appropriate cell types, tyrosinase and extracellular superoxide dismutase 3 (SOD3) [74, 75, 85]. Through these substrates, ATP7A contributes to extracellular-matrix remodelling, connective-tissue integrity, pigmentation, and antioxidant defence. ATP7B has a distinct predominant role in hepatocytes, where it transfers copper into the secretory pathway for incorporation into ceruloplasmin and supports systemic copper and iron homeostasis [22, 42]. Thus, the biological consequences of ATP7A or ATP7B activity depend on tissue distribution, intracellular trafficking, and the specific cuproenzymes supplied.

Copper also plays a role within the nucleus, helping to control gene transcription, in addition to its roles in cytoplasmic and mitochondrial physiology [50]. For instance, in HepG2 cells exposed to hypoxia, adequate intracellular copper and CCS are required for full hypoxic inducible factor-1 (HIF-1) activation: copper chelation with tetraethylenepentamine (TEPA) or CCS knockdown suppresses hypoxia-induced HIF-1 activity, whereas copper supplementation reverses the effect of chelation but not the effect of CCS silencing [84]. Mechanistically, copper depletion does not primarily prevent HIF-1α protein accumulation or stability; rather, it reduces HIF-1α binding to hypoxia-response elements (HREs) in target genes and weakens recruitment of the transcriptional coactivator p300, thereby impairing assembly of the HIF-1 transcriptional complex [86]. Later work further showed that copper can regulate HIF-1α target-gene selectivity by altering HIF-1α occupancy at specific HREs, for example affecting BNIP3 but not IGF2 promoter binding under low-copper conditions [87]. Therefore, the copper–CCS axis should be understood not only as a regulator of HIF-1α expression, but also as a facilitator of HIF-1α DNA binding and coactivator recruitment during hypoxic transcriptional responses. ATOX1 has also been reported to translocate to the nucleus and to promote the expression of proliferation- and angiogenesis-associated genes, including CCND1, in selected cellular models [92]. However, direct DNA binding by human ATOX1 has not been consistently reproduced in vitro, and its nuclear transcriptional mechanism remains incompletely resolved [88, 89].

## Dysregulation of copper homeostasis in tumours

Tumour cells can adjust their intrinsic copper equilibrium to meet their requirements for rapid cell growth, a condition newly labelled as cuproplasia or copper-induced cell proliferation [90].

### Cuproplasia and copper reprogramming

Many malignant cells exhibit an increased demand for copper, although the mechanisms used to satisfy this demand vary among tumour types. Increased CTR1/SLC31A1 expression can enhance copper influx, whereas changes in ATP7A, ATP7B, copper chaperones, metallothioneins, or vesicular trafficking may alter copper efflux, sequestration, or delivery to specific copper-dependent pathways [91]. These changes should not be assumed to increase intracellular copper solely on the basis of transporter expression, because ATP7A and ATP7B can promote copper export or excretion when correctly trafficked. Upregulation of CTR1/SLC31A1 has been associated with increased copper uptake in experimental models of colorectal and pancreatic cancer [46, 92]. After cellular entry, copper can be directed toward secretory, mitochondrial, or nuclear pathways. For example, ATP7A delivers copper to LOX/LOXL enzymes and thereby promotes tumour growth and metastatic progression in experimental models, whereas copper-bound ATOX1 can support proliferation-associated transcription [30, 89]. Systemic changes may also contribute, because increased serum copper and/or ceruloplasmin have been reported in several cancer cohorts, although these findings are not uniform across all studies and may partly reflect inflammatory responses [93–97].

As one example, melanoma cells containing the BRAF^V600E^ mutation rely on copper to activate the MAPK pathway, because copper directly binds to MEK1/2, facilitating kinase activity. Reducing copper levels using chelators like ammonium tetra-thiomolybdate or deleting the copper transporter CTR1 inhibited MEK1/2 activity and effectively reduced BRAF^V600E^ tumour growth [98]. Likewise, the copper chaperone ATOX1 can deliver copper ions to the nucleus to act as a transcription factor. ATOX1 upregulates the expression of cyclin D1, thus promoting cell cycle progression and proliferation, particularly in mutant p53 cancer cells [91]. Several cancer types have higher labile copper pools combined with increased ATOX1 expression, and this fact makes them vulnerable when copper is chelated [98, 99]. Therefore, by reprogramming copper intake and transport, cancers can generate a pro-proliferation environment called cuproplasia, where copper-dependent proteins and signalling pathways support aberrant cell growth.

### Copper and angiogenesis

Copper can promote tumour angiogenesis by supporting hypoxia-responsive transcription, growth-factor signalling, and the activity of copper-dependent extracellular enzymes. In experimental cancer-cell models, copper increased HIF-1α, GPER, and VEGF expression through EGFR–ERK–c-Fos signalling [100]. Conditioned media from copper-treated tumour cells consequently promoted endothelial-cell migration, proliferation, and tube formation. Conversely, copper chelation reduced HIF-1/VEGF signalling and inhibited angiogenic responses in selected cellular and animal models [100]. Copper also supports the activity of lysyl oxidase-family enzymes, which remodel the extracellular matrix and can influence vascular organisation and tumour invasion. These effects do not imply that every tumour is globally hypoxic or uniformly copper-dependent. Copper should therefore be described as a context-dependent modulator of hypoxia-linked and extracellular-matrix-associated angiogenic pathways.

This relationship should not be interpreted as meaning that all tumours are uniformly hypoxic. Many solid tumours contain functional and sometimes hypervascular regions, but tumour vessels are often structurally and functionally abnormal, causing oxygen delivery to vary across short distances and over time. Therefore, relatively oxygenated and poorly oxygenated microregions can coexist within the same tumour [101, 102]. In this context, local copper availability may intersect with microregional hypoxia by supporting HIF-1 transcriptional activity in oxygen-stressed regions and by promoting proangiogenic signalling. Consistent with this model, copper sulfate increased HIF-1α, GPER, and VEGF expression in breast and hepatic cancer cells through EGFR–ERK–c-Fos signalling, linking copper availability to VEGF-mediated angiogenic responses [100]. Thus, copper should be presented as a context-dependent modulator of hypoxia-linked angiogenesis, rather than as evidence that all tumour models discussed are globally hypoxic.

### Copper and metastasis

Copper tends to enhance the ability of tumours to proliferate and spread throughout the body. One example is lysyl oxidase (LOX) and LOX-like proteins (LOXL1-4), which are copper-dependent amine oxidases that catalyse the covalent cross-linking of collagen and elastin fibres in the extracellular matrix (ECM). By promoting the formation of these cross-links, LOX enzymes harden, stabilise, and remodel the ECM, which is important for cancer metastasis. Low-oxygen malignant tumours frequently overproduce LOX, which cross-links collagen and elastin chains to produce a thick and tough matrix substrate that acts as a "soil" for metastatic "seeds". In experimental tumour models, ATP7A supplies copper to LOX and LOXL enzymes within the secretory pathway, supporting their enzymatic maturation and promoting extracellular-matrix remodelling, tumour growth, and metastasis [30]. This function reflects copper redistribution to secretory cuproenzymes rather than a general increase in the total intracellular copper pool. If copper supplies are low, the LOX proteins fail to operate efficiently, thus impeding tumour spread. Consistent with this mechanism, copper chelation or genetic disruption of ATP7A-dependent copper delivery reduced LOX-family activity, collagen cross-linking, and metastatic dissemination in experimental models, with comparatively limited effects on primary-tumour growth [30, 103].

Clinical studies have associated increased LOX-family expression with aggressive behaviour in several malignancies. However, expression-based associations do not by themselves establish copper dependence or causality. Other extracellular proteases, including matrix metalloproteinases and components of the plasminogen-activation system, also contribute to invasion and metastasis, but they should not be described as copper-dependent proteins unless direct biochemical evidence supports that classification. Overall, the strongest established copper-dependent mechanism in this context is ATP7A-mediated copper delivery to LOX-family enzymes.

### Immune effects and DNA damage

An underappreciated effect of the copper imbalance within tumours is the way it alters the immunological environment, allowing tumours to avoid immunological monitoring and attack. Significant amounts of copper inside the tumour, as well as the surrounding tissue, can switch immune system cells to an immunosuppressive phenotype [104]. A recent study by Gong et al., demonstrated the potential of cuproptosis-related non-coding RNAs in immunotherapy of squamous cell carcinoma, which highlights the potential of cuproptosis in immunotherapeutic-based interventions [105]. For example, myeloid-derived suppressor cells (MDSCs) tend to require copper for their optimal viability and function. In an animal experiment, a copper-chelating drug reduced the number of myeloid-derived suppressor cells through apoptotic cellular death while also boosting T-cell anti-tumour activity, suggesting that copper can cause immune suppression inside tumours [106]. Excess copper has also been linked to cancer development. Unbound Cu ions (Cu^+^ or Cu^++^) are capable of initiating Fenton-like reactions to create hydroxyl radicals, and causing damage to DNA through oxidation and genome-wide dysfunction in tumours [17]. Wilson disease provides a clinical example of chronic tissue copper accumulation caused by defective ATP7B-mediated biliary copper excretion. Hepatocellular carcinoma has been reported in patients with Wilson disease, particularly in the setting of chronic liver injury or cirrhosis; however, the available evidence does not by itself establish that Wilson disease uniformly increases hepatocellular-carcinoma incidence or that copper accumulation directly causes malignant transformation [107, 108]. Therefore, Wilson disease illustrates the pathological consequences of sustained hepatic copper accumulation but should not be presented as direct proof of copper-induced carcinogenesis.

More broadly, as tumours progress, dysregulated copper metabolism may increasingly support malignant growth, and elevated tumour copper levels have been associated with more aggressive disease. Copper powers essential processes that range from ATP production to antioxidant protection [109]. A copper imbalance has been linked to tumour growth-promoting pathways. For instance, copper can trigger vascular growth and angiogenic mediators (such as vascular endothelial growth factor—VEGF) as well as specific receptor tyrosine kinases, leading to loss of tumour dependence on intrinsic growth factors [110]. In conclusion, abnormal copper metabolism contributes to tumour development through multiple mechanisms, highlighting its potential as a therapeutic target in cancer management.

## Cuproptosis and its molecular pathways

### Discovery and definition of cuproptosis

Copper has long been recognised as a double-edged sword in biology—essential in trace amounts but toxic in excess [50, 111, 112]. By the late twentieth century, studies showed that abnormally high copper levels could damage cells and even accelerate tumour cell death [50, 113]. These early observations hinted that copper might be able to trigger a unique cell death process, going beyond generalised oxidative damage. This possibility remained speculative until very recently. In 2022, Tsvetkov and colleagues formally identified and named “cuproptosis” as a distinct form of regulated cell death driven by copper [4]. Cuproptosis was defined as a copper-dependent, non-apoptotic cell death mechanism, mechanistically separate from other known pathways like apoptosis, ferroptosis, and necroptosis [4, 114, 115].

In the Tsvetkov et al. study, excess copper was demonstrated to lethally disrupt mitochondrial function, highlighting that mitochondria are the central organelle in cuproptosis [4]. Subsequent research confirmed that cuproptosis occurred predominantly in cells reliant on oxidative phosphorylation, where mitochondrial respiration is active, while purely glycolytic cells are comparatively resistant [50, 114]. Importantly, cuproptosis does not show the classical hallmarks of apoptosis or other canonical death pathways; For example, inhibiting caspases or ferroptosis fails to rescue copper-challenged cells, suggesting a fundamentally novel mode of cell death [4].

Cuproptosis should therefore be defined by copper dependence, mitochondrial respiratory dependence, and involvement of lipoylated mitochondrial proteins. Copper accumulation, reactive oxygen species generation, or loss of viability alone is insufficient to identify a phenotype as cuproptosis [50, 114, 116].

### Copper import and mitochondrial accumulation

The execution of the cuproptosis process begins with the uptake and localisation of copper within the cell, especially into mitochondria. Under normal conditions, extracellular Cu^+^ ions are imported through high-affinity copper transporter 1 (CTR1, also known as SLC31A1) on the plasma membrane [114]. Once inside, copper is chaperoned by molecules like glutathione and metallothioneins or transferred to dedicated copper-binding proteins. These homeostatic pathways ordinarily ensure that copper is safely sequestered or incorporated into essential enzymes [50, 114]. However, when copper levels overwhelm the cellular buffering capacity, excess copper ions can be abnormally trafficked into the mitochondria. Copper may reach the mitochondrial matrix via copper-binding carriers and possibly via the phosphate transporter SLC25A3, which has been implicated in mitochondrial copper import [114].

To produce cuproptosis-inducing conditions, this mitochondrial copper overload can be experimentally achieved by using copper ionophores (small molecules that ferry copper across membranes). Notably, the anticancer compounds elesclomol (ES) and disulfiram (DSF) are in fact copper ionophores that drive copper accumulation in mitochondria [117–119]. ES was shown to deliver Cu^2+^ into the mitochondrial compartment, a process crucial for triggering cuproptotic death [118]. As a result of enhanced Cu import or ionophore-mediated delivery, an excessive pool of copper accumulates inside the mitochondrial matrix. This mislocalized copper is the catalyst for cuproptosis. The mitochondrion, normally involved in metabolic energy production, becomes the site where copper cytotoxicity is unleashed. The mere presence of excess mitochondrial copper is dangerous. It further sets off a cascade of molecular events described below that ultimately commit the cell to dying [4]. Mitochondrial copper accumulation alone does not establish cuproptosis. The decisive event is the interaction of bioavailable copper with an intact lipoylated-protein pathway in a metabolically susceptible cell. Accordingly, copper-dependent loss of viability should be classified as cuproptosis only when supported by evidence such as FDX1 or lipoylation dependence, lipoylated-protein aggregation, iron–sulfur cluster protein loss, and mitochondrial respiratory dependence.

### The FDX1-lipoic acid pathway and lipoylated TCA enzymes

**Fig. 3:**
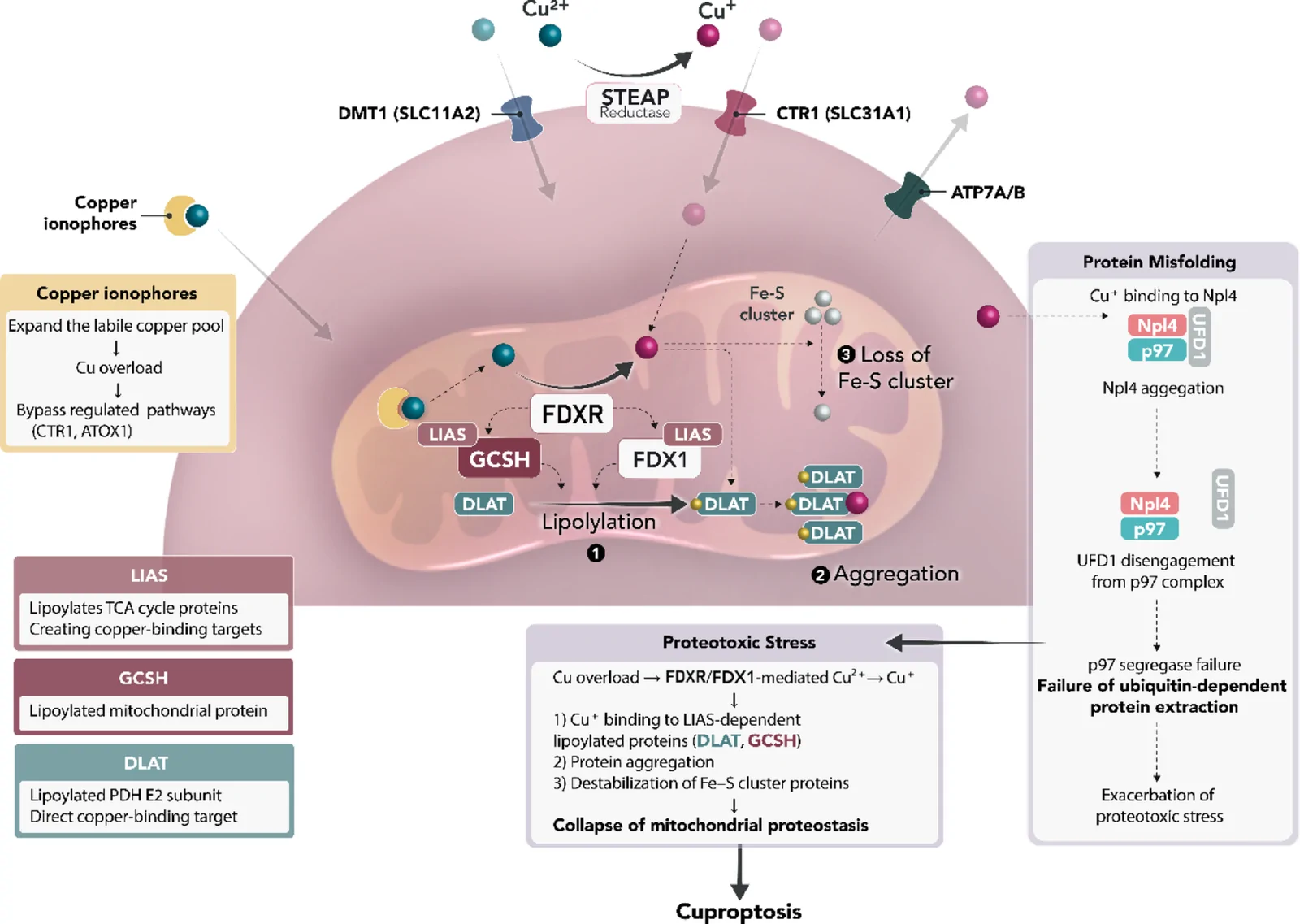
Mechanistic overview of cuproptosis: copper overload, mitochondrial protein lipoylation, and proteotoxic stress. Key molecular mechanisms driving **cuproptosis** are triggered by excess intracellular copper. Copper ionophores increase the intracellularly available copper pool by transporting copper across cellular membranes or redistributing copper independently of the normal CTR1-mediated uptake route. This pharmacological process may also overwhelm buffering, chaperoning, and ATP7A- or ATP7B-dependent copper-export mechanisms. Within the mitochondria, Cu⁺ binds to lipoylated proteins in the tricarboxylic acid (TCA) cycle, especially DLAT (dihydrolipoamide S-acetyltransferase) and GCSH, both of which are modified by LIAS (lipoic acid synthase). This Cu⁺-dependent binding is facilitated by the FDXR–FDX1 mitochondrial electron-transfer system, in which FDXR (ferredoxin reductase) provides electrons from NADPH to FDX1. Copper binding induces aggregation of lipoylated proteins, destabilisation of Fe–S clusters, and collapse of mitochondrial proteostasis. Concurrently, excess Cu⁺ interacts with Npl4, a component of the p97/VCP complex, disrupting ubiquitin-dependent protein extraction and aggravating proteotoxic stress. The culmination of these events results in mitochondrial dysfunction and cell death via cuproptosis. CTR1 (SLC31A1): Copper Transporter 1, DMT1 (SLC11A2): Divalent Metal Transporter 1, STEAP: Six Transmembrane Epithelial Antigen of the Prostate, ATP7A/B: ATPase copper transporters, FDXR: Ferredoxin Reductase, FDX1: Ferredoxin 1, LIAS: Lipoic Acid Synthase, DLAT: Dihydrolipoamide S-acetyltransferase, GCSH: Glycine Cleavage System Protein H, Fe–S: Iron–Sulfur, TCA cycle: Tricarboxylic Acid Cycle, Npl4: Nuclear Protein Localization Protein 4, p97: AAA ATPase (also known as VCP), UFD1: Ubiquitin Fusion Degradation Protein 1, UPRmt: Mitochondrial Unfolded Protein Response

One pivotal insight from the discovery of cuproptosis was the identification of specific mitochondrial proteins that render cells sensitive to copper. Foremost among these is ferredoxin 1 (FDX1), a mitochondrial ferredoxin that emerged from genome-wide screens as an essential regulator of cuproptosis [4] (Fig. 3). FDX1 is a mitochondrial [2Fe–2S] ferredoxin and an essential regulator of Cuproptosis with several potential functional roles, among which the reduction of Cu^2^⁺ represents a particularly important process. In mammalian mitochondria, however, this Cu^2^⁺-reducing activity is not mediated by FDX1 alone, but is closely linked to FDXR through the mitochondrial FDXR–FDX1 electron-transfer system. In this system, FDXR receives electrons from NADPH and transfers them to FDX1, which supports mitochondrial copper redox cycling and contributes to the generation of Cu⁺ species that can bind lipoylated TCA-cycle proteins under copper-overload conditions [4, 114, 120]. In parallel, FDX1 plays an interesting role in protein lipoylation. Under normal conditions, FDX1 helps maintain the activity of the lipoic acid synthase complex that binds the lipoic acid cofactor to specific enzymes in the TCA cycle [114]. Indeed, FDX1 has been shown to physically interact with lipoic acid synthase (LIAS), facilitating the lipoylation of target proteins [5]. This is significant because the key factors in cuproptosis are lipoylated mitochondrial enzymes. Tsvetkov et al. discovered that copper selectively binds to lipoylated TCA-cycle enzymes, leading to their abnormal oligomerisation and aggregation [4].

Among the mitochondrial proteins functionally influenced by FDX1, dihydrolipoamide S-acetyltransferase (DLAT), the E2 component of the pyruvate dehydrogenase complex, is particularly important. DLAT normally carries a lipoic acid moiety required for its enzymatic activity in the TCA cycle. However, in cuproptosis, the FDXR–FDX1 electron-transfer system may support mitochondrial copper reduction and cuproptosis susceptibility. FDX1 also regulates protein lipoylation through its interaction with LIAS. Together, these functions connect mitochondrial copper redox biology with the abundance of lipoylated proteins that serve as copper-binding targets, triggering its aggregation into large complexes [4, 50, 114]. This phenomenon is not limited to DLAT alone, and other mitochondrial enzymes can be lipoylated (such as components of α-ketoglutarate dehydrogenase and related complexes), which are also presumed to be targets of copper binding [114]. The net effect is a collapse in critical TCA-cycle enzyme functionality due to protein aggregation. Genetic ablation studies reinforced the centrality of this pathway. Deleting FDX1 or disabling the lipoic acid assembly line (e.g., knockout of LIAS or the lipoyl transferase LIPT1) protected cells from cuproptosis by preventing the copper-protein interactions from occurring [4, 114]. Thus, the FDX1-lipoic acid axis forms the core of the cuproptotic mechanism. It creates an Achilles’ heel that copper can exploit to kill cells. This is a set of lipoylated mitochondrial proteins whose copper-mediated aggregation leads to a toxic gain-of-function (proteotoxic clusters) and a loss-of-function in metabolism. This precisely orchestrated failure of mitochondrial protein homeostasis is the defining molecular signature of cuproptosis [63, 121].

### Loss of iron–sulfur (Fe–S) clusters, proteotoxic stress, and cell death execution

Copper binding to lipoylated mitochondrial proteins is accompanied by the loss or destabilisation of iron–sulfur-cluster proteins, constituting a second major molecular feature of cuproptosis [4]. Because iron–sulfur proteins support mitochondrial respiration, metabolic enzyme activity, and redox regulation, their depletion is expected to amplify mitochondrial dysfunction. However, the precise molecular process responsible for their loss during cuproptosis remains incompletely defined and should not be attributed specifically to copper-mediated iron displacement without direct experimental evidence. Aggregation of lipoylated proteins together with iron–sulfur-cluster protein loss imposes substantial mitochondrial proteotoxic stress. These changes disrupt the function of lipoylated metabolic complexes and impair mitochondrial homeostasis, ultimately producing copper-dependent cell death in metabolically susceptible cells [4]. Reactive oxygen species may accompany copper overload in some experimental systems, but oxidative stress is neither sufficient to define cuproptosis nor established as its principal execution mechanism.

Disulfiram-derived copper complexes can disrupt protein homeostasis through a mechanism that is distinct from the canonical FDX1- and protein-lipoylation-dependent cuproptosis pathway. In cells treated with disulfiram and copper, the copper-containing metabolite CuET induces aggregation and immobilisation of NPL4, an adaptor of the p97/VCP segregase complex, thereby impairing the processing of ubiquitinated proteins and increasing proteotoxic stress [122, 123]. Although this mechanism may contribute to the anticancer effects of disulfiram–copper combinations, current evidence does not establish NPL4 aggregation as a core execution mechanism of cuproptosis. It should therefore be described as a separate copper-associated proteotoxic pathway unless future studies demonstrate direct dependence on FDX1, mitochondrial protein lipoylation, or other defining components of cuproptosis.

Ultimately, the convergence of these events, including enzymatic inhibition (from Fe-S cluster loss), energy crisis, oxidative stress, and protein aggregation with impaired clearance, irreversibly pushes the cell to death [50]. Morphologically, cuproptotic cell death is distinguished by extensive protein aggregation and mitochondrial swelling, rather than DNA fragmentation or membrane rupture, consistent with its unique biochemical execution mechanism [4, 114]. This multi-factorial disruption of essential cellular homeostasis underlies the irreversible nature of cuproptosis once initiated.

### Crosstalk with other regulated cell death pathways

#### Ferroptosis and apoptosis

Cuproptosis does not occur in isolation from other cell death processes, and recent work has begun to map its relationship with other forms of regulated cell death. Intriguingly, there is evidence of both overlap and independence between cuproptosis and pathways like apoptosis and ferroptosis [4, 50]. One point of convergence is the generation of ROS: excess copper can catalyse Fenton-like reactions that produce ROS, which in turn can initiate classical apoptosis cascades [124]. In fact, early studies using copper ionophores reported some apoptotic features occurring in cancer cells. For example, disulfiram-copper complexes were shown to trigger high ROS levels and activate caspase-dependent extrinsic apoptosis in melanoma and leukaemia cells [125, 126]. This suggested that in some contexts, copper overload could simultaneously engage apoptotic signalling. However, the defining studies into cuproptosis demonstrated a critical difference, because applying canonical apoptosis inhibitors (e.g., caspase inhibitors) did not prevent copper-induced death in cells undergoing cuproptosis [4]. Thus, while cuproptosis may generate pro-apoptotic signals such as ROS, its execution bypasses the traditional apoptotic machinery, indicating that the copper-initiated cell death is a fundamentally distinct pathway [50]. Another possible interplay is seen with ferroptosis, the iron-dependent lipid peroxidation-mediated form of cell death. Cuproptosis and ferroptosis share a common trigger in that both can be potentiated by glutathione depletion or ROS accumulation.

Recent studies have revealed a bidirectional interaction between these pathways. On one hand, classical ferroptosis inducers such as erastin and sorafenib were found to amplify cuproptosis in cancer cells by upregulating FDX1 and promoting the aggregation of lipoylated proteins [63]. This implied that driving cells into an oxidative, iron-dependent stress state could make them more susceptible to copper cytotoxicity. On the other hand, copper can promote ferroptosis under certain conditions. Xue et al. showed that copper overload in pancreatic carcinoma cells triggered the autophagic degradation of GPX4 (a key ferroptosis-defence enzyme), thereby exacerbating erastin-induced ferroptotic cell death [127]. Moreover, both processes are linked to mitochondria. Mitochondrial metabolic activity promoted cysteine-deprivation ferroptosis (through ROS generation), and the organelle was central to cuproptosis [114]. These findings point to a partial convergence of cuproptosis and ferroptosis at the level of upstream metabolic stress and glutathione/ROS balance. Indeed, high copper levels can deplete glutathione and produce lipid peroxides, fulfilling conditions for ferroptosis initiation [127]. Despite these interactions, cuproptosis maintains its distinct identity.

Notably, treatment with ferrostatin-1 (a ferroptosis inhibitor) failed to rescue cells from cuproptotic death induced by ES-copper, showing that the end stages of the cuproptosis pathway were separate from ferroptotic execution [4]. Beyond apoptosis and ferroptosis, copper stress can affect other cell death and survival pathways as well. For instance, cells can initiate autophagy in response to copper accumulation. Autophagy may have a dual role in copper-stressed cells: it may protect cells by removing damaged mitochondria and aggregated proteins, or it may contribute to cell death when activated excessively [128–130].

#### Pyroptosis

Evidence directly linking cuproptosis to pyroptosis remains preliminary. Nanomedicine studies have reported tumour treatments designed to activate both pathways, but simultaneous detection of cuproptosis-associated and pyroptosis-associated markers does not establish direct mechanistic crosstalk [131]. In hepatocytes expressing the Wilson-disease-associated ATP7B R778L variant, chronic copper exposure activated autophagy and suppressed necroptotic signalling, thereby increasing cellular tolerance to copper toxicity [132]. This model demonstrates an adaptive response to copper accumulation caused by dysfunctional ATP7B; it does not establish that the observed cell death or survival phenotype represents cuproptosis. There are also emerging links between cuproptosis and pyroptosis (inflammatory cell death), especially in cancer immunotherapy treatments [133]. For example, novel nanoparticles have been designed to induce both cuproptosis and pyroptosis in tumours to synergistically boost anti-tumour immunity [134]. While such approaches are still experimental, they highlight the potential for cross-communication between copper-induced death and immune-mediated anti-tumour pathways [50, 114].

### Tumour-type specificity, biomarkers, and therapeutic implications

Although cuproptosis provides a general mechanistic framework, current evidence suggests that susceptibility is not uniform across all cancers and is likely to be greatest in tumours that combine altered copper handling, mitochondrial respiration dependence, and intact lipoylated TCA-cycle machinery. In the original cuproptosis study, copper-dependent cell death required mitochondrial respiration and occurred through copper binding to lipoylated TCA-cycle proteins, followed by lipoylated protein aggregation, Fe–S cluster protein loss, proteotoxic stress, and cell death [4]. BRAF-driven melanoma and related BRAF-mutant tumours provide one example of copper-dependent oncogenic signalling, because CTR1 loss or disruption of copper binding to MEK1 reduced BRAF^V600E^-driven MAPK signalling and tumour growth, consistent with findings that copper chelators reduced the growth of BRAF^V600E-transformed and BRAF-inhibitor-resistant tumour cells [98]. In clear cell renal cell carcinoma, advanced tumours accumulate copper and allocate it to cytochrome c oxidase, supporting bioenergetic, biosynthetic, and redox remodelling that promotes tumour progression [109]. In hepatocellular carcinoma, recent evidence suggests a more direct vulnerability to cuproptosis. One study showed that elesclomol–copper induced DLAT oligomerisation and FDX1-dependent cuproptotic cell death, and p53 enhanced cuproptosis sensitivity through FDXR-mediated FDX1 upregulation [7]. Copper-mediated SEC14L3 signalling also promoted FDX1 expression, DLAT lipoylation/oligomerisation, and suppression of HCC growth in vitro and in vivo, further supporting HCC as a tumour type in which the FDX1–DLAT axis may be therapeutically relevant [135]. Additional tumour-specific evidence includes ovarian cancer, where elevated levels of intracellular copper resulted in cuproptosis which inhibited tumour progression and enhanced cisplatin toxicity in vitro and in vivo, and prostate cancer, where increasing intracellular bioavailable copper selectively killed cancerous prostate cells in vitro and in vivo [9, 136]. Breast cancer may be particularly relevant to copper-depletion and copper-homeostasis-disruption strategies, because tetrathiomolybdate-based copper depletion has been tested in high-risk breast cancer and preclinical lung-metastasis models, while more recent work suggests that disruption of NF-κB-mediated copper homeostasis can sensitize breast cancer to cuproptosis [10, 103]. NF-κB also regulates other cell-death pathways, including apoptosis [137]. Together, these tumour-specific findings suggest that sensitivity to cuproptosis-based therapy is unlikely to depend on a single marker, but rather on an integrated profile of copper handling, mitochondrial metabolism, and intact lipoylation machinery. Candidate biomarkers should be considered as a multidimensional profile rather than as individual expression markers. Relevant features may include: (i) CTR1/SLC31A1-mediated copper uptake; (ii) ATP7A and ATP7B expression, localisation, and trafficking, evaluated separately; (iii) FDX1, FDXR, LIAS, LIPT1, DLAT, and other components of the protein-lipoylation pathway; (iv) mitochondrial respiratory dependence; and (v) direct measurements of tumour copper distribution and bioavailability. Expression data alone are insufficient to determine copper flux, transporter activity, or cuproptosis competence.

In summary, cuproptosis can be distinguished from other regulated cell death pathways by its unique molecular mechanism, but it does not occur in a vacuum. Copper’s impact on the cellular redox state and protein homeostasis means that cuproptosis can show features associated with apoptosis (ROS generation) or ferroptosis (GSH depletion, lipid peroxidation) [50]. What decisively differentiates cuproptosis is the central requirement for copper and mitochondrial protein lipoylation, which is not seen in other cell death pathways. As research continues, understanding how cuproptosis overlaps with, or diverges from, pathways like ferroptosis and apoptosis will be crucial. Such insights could reveal opportunities to co-target these pathways: for example, combining a copper ionophore with a ferroptosis inducer, or using autophagy inhibitors to tip the balance towards cell death in copper-loaded tumours [63, 127]. Ultimately, mapping the crosstalk between cuproptosis and other cell death networks will enhance our ability to manipulate these processes for therapeutic benefit, ensuring that cancer cells have no escape route from destruction [4].

## Conclusion

Collectively, the evidence reviewed here establishes copper as more than an essential micronutrient in cancer biology. Its tightly regulated trafficking, mitochondrial handling, and redox activity shape tumour growth, angiogenesis, metastasis, and stress adaptation, while disruption of this balance can expose a selective vulnerability. Cuproptosis forms a mechanistic framework for this vulnerability by linking copper overload to mitochondrial respiration, lipoylated TCA-cycle proteins, FDX1-dependent copper redox biology, Fe–S cluster destabilisation, and proteotoxic collapse. This pathway is distinct from, but functionally connected to, apoptosis, ferroptosis, pyroptosis, and autophagy, suggesting that copper-induced death should be viewed within a broader network of metabolic and stress-response programmes. Therapeutically, copper ionophores, chelators, transporter modulation, metabolic rewiring, and nanomedicine-based delivery systems offer complementary approaches to manipulate copper homeostasis. However, clinical translation will require careful tumour stratification, robust biomarkers of cuproptosis competence, and strategies that maximise cancer-selective copper toxicity while preserving physiological copper-dependent functions. Future studies should define context-specific resistance mechanisms, clarify immune consequences of cuproptosis, and validate combination regimens in clinically relevant models. A deeper understanding of copper biology may therefore transform cuproptosis from a recently described molecular phenomenon into a rationally exploitable axis for cancer therapy.

## Data Availability

No datasets were generated or analysed during the current study.

## Materials availability

Not applicable. No new biological materials, cell lines, animal models, software, or experimental reagents were generated for this review.
